# Delayed Presentation of Arterioportal Hepatic Fistula in the Absence of Portal Hypertension: A Case Report

**DOI:** 10.7759/cureus.117969

**Published:** 2026-10-15

**Authors:** Matthew J Scearbo, Regina Wilson, Trisha Patel, Alejandro Biglione

**Affiliations:** 1 College of Medicine, Nova Southeastern University Dr. Kiran C. Patel College of Osteopathic Medicine, Tampa, USA; 2 Internal Medicine, Wellington Regional Medical Center, Wellington, USA; 3 College of Medicine, Nova Southeastern University Dr. Kiran C. Patel College of Osteopathic Medicine, Davie, USA

**Keywords:** abdominal ct angiography, arterioportal fistula, image-guided (ir) embolization, percutaneous liver biopsy, portal hypertension

## Abstract

An arterioportal hepatic fistula (APF) is an abnormal vascular communication between a branch of the hepatic artery and the portal vein. APFs most often occur secondary to trauma but can present due to hepatic procedures, abdominal neoplasm, or congenital malformation. APFs are often asymptomatic due to being small in size and located in anatomically insignificant areas. Large or centrally located APFs often present with portal hypertension, hemorrhage, or hepatic dysfunction. This case report describes a 79-year-old woman who developed a symptomatic centrally located APF without portal hypertension sequelae after a liver biopsy. The incidence, pathophysiologic mechanisms, presentation, diagnosis, and management are discussed.

## Introduction

An arterioportal hepatic fistula (APF) is an abnormal vascular communication between a branch of the hepatic artery and the portal vein. Etiologies of APFs, in order of most common, include trauma, iatrogenic procedures, congenital malformations, tumors, and hepatic artery aneurysms [1]. Although uncommon, APF is a well-described complication of liver biopsy occurring in 4-5% of cases, likely due to the close anatomic relationship of the portal vein and hepatic artery within the portal triad. Common complications of APF include portal hypertension, gastrointestinal bleeding, and ascites [2,3].

This case report presents the case of a 79-year-old female with a history of nonalcoholic steatohepatitis (NASH), diabetes mellitus, and hypertension, who underwent an outpatient percutaneous liver biopsy to evaluate elevated liver enzymes. Three weeks later, she presented with severe right-sided abdominal and back pain radiating anteriorly. The patient denied nausea, vomiting, or gastrointestinal bleeding. Imaging revealed a centrally located APF, for which the patient's presentation is atypical. The incidence, pathophysiologic mechanisms, presentation, diagnosis, and management are discussed.

## Case presentation

The patient was a 79-year-old female with a history of nonalcoholic steatohepatitis who underwent outpatient percutaneous liver biopsy to evaluate elevated liver enzymes. Three weeks later, she presented with severe right-sided abdominal pain. The patient denied nausea, vomiting, or gastrointestinal bleeding. 

Her workup included a CT scan that demonstrated a 2.5 x 2.0 x 5.0 cm liver hematoma at the biopsy site with associated abnormal vascularity suspicious for an arterio-portal fistula. Further evaluation with CT angiography and mesenteric angiogram confirmed the presence of a hepatic segment 8 APF, as shown in Figures 1-2.

**Figure 1:**
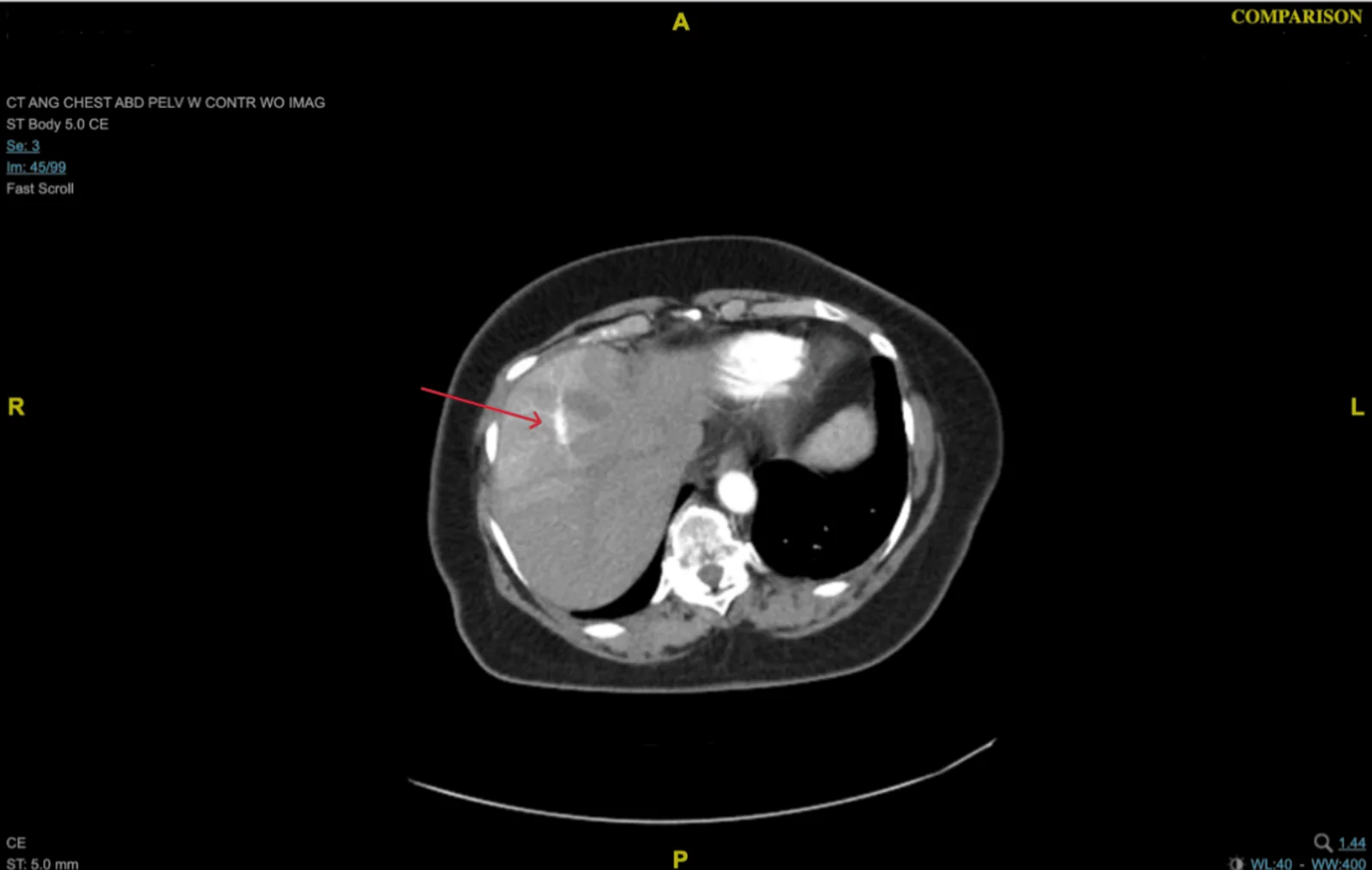
CT angiography of the abdomen with IV contrast with axial reconstruction The red arrow points to the arterioportal hepatic fistula.

**Figure 2:**
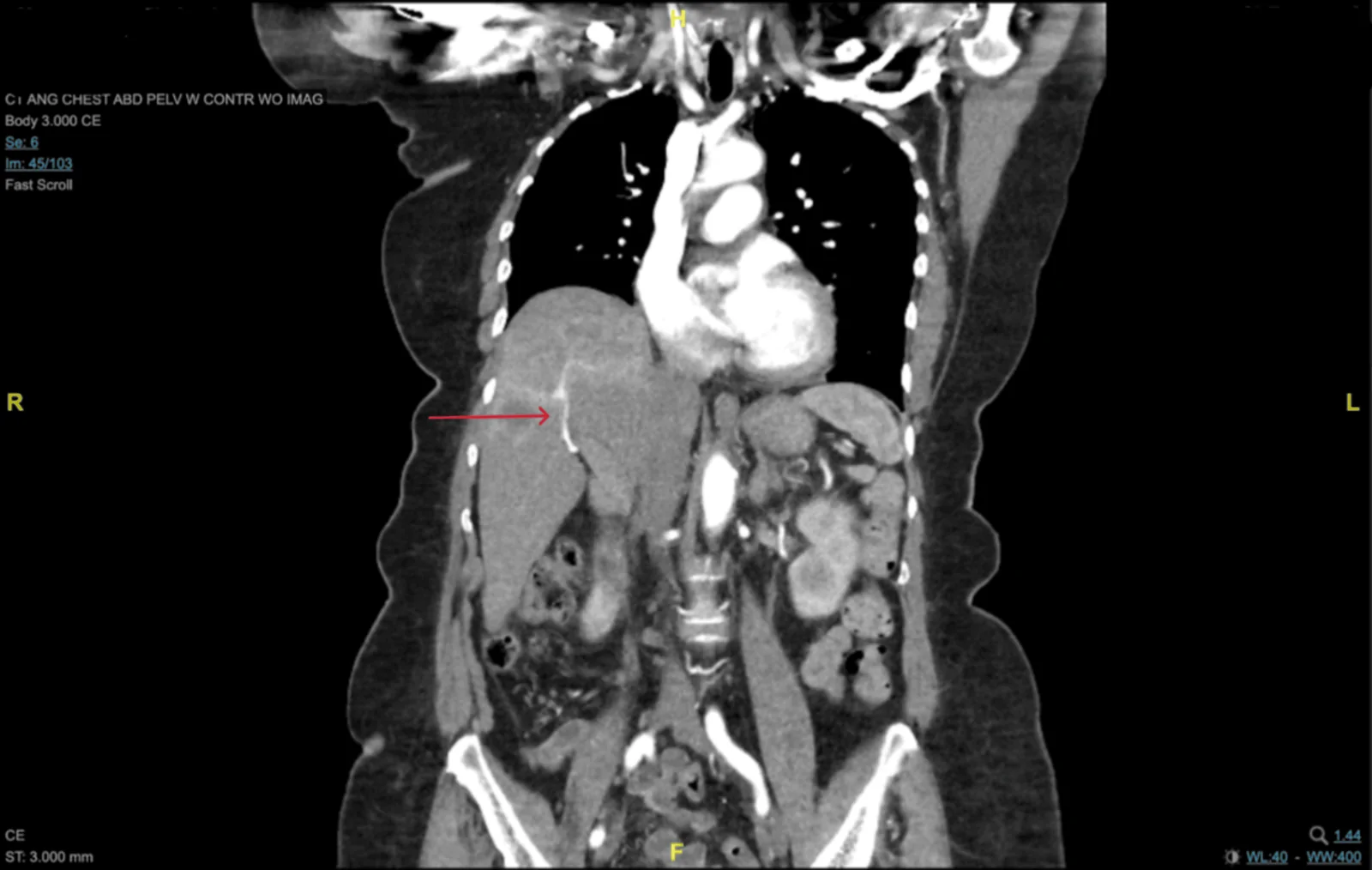
CT angiography of the abdomen with IV contrast with coronal reconstruction The red arrow points to the arterioportal hepatic fistula.

Interventional radiology (IR) was consulted. The patient underwent ultrasound-guided right femoral artery access, mesenteric angiography, and selective catheterization of the segment 8 artery using a microcatheter system. The fistula was embolized using two 5 x 10 mm Penumbra Ruby Coils. Post-embolization angiography confirmed complete closure of the fistula. The patient tolerated the procedure well and reported significant improvement in her abdominal pain. Follow-up imaging demonstrated normalization of hepatic vascular flow and no further fistula recurrence.

## Discussion

APFs are categorized based on their size, location, and hemodynamic significance. Type 1 APFs are small, in the periphery of the hepatic vasculature, and most often asymptomatic without hemodynamic significance. The vast majority of APFs following liver biopsy are clinically silent and fall into the type 1 category. Type 2 APFs are either large or central in location within the hepatic vasculature, hence causing portal hypertension and hepatic parenchymal damage [4]. Based on current literature, type 2 APFs should present with significant complications, such as portal hypertension, ascites, variceal bleeding, hepatic vein thrombosis, mesenteric steal syndrome, and other changes in the hepatic parenchyma [5]. Hepatic segment 8 is located centrally in the superior aspect of the liver; hence, the APF described in this case would be categorized as type 2. This presentation of a type 2 APF is atypical, lacking any signs of portal hypertension, ascites, or variceal bleeding. While APFs can present with right upper quadrant (RUQ) pain, it is typically attributed to the presence of the complications of portal hypertension. Therefore, this patient’s RUQ pain was more likely due to the presence of a moderate-sized hematoma, rather than the hepatic pathology that would be expected with type 2 APF. This case suggests that the presence of portal hypertension and its sequelae may be unreliable markers for the presence of APF, even those that are large and centrally located. Thus, it is important to consider this complication following invasive hepatic procedures regardless of presentation. 

This case highlights a centrally located (type 2) APF without the expected clinical manifestations of portal hypertension, underscoring that symptom severity and fistula classification may not always correlate. Clinicians should maintain a high index of suspicion for vascular complications in patients presenting with persistent or unexplained abdominal pain following hepatic instrumentation, even in the absence of classic sequelae. Prompt imaging with contrast-enhanced CT and multidisciplinary management with interventional radiology is essential to prevent progression to more serious complications.

## Conclusions

APFs should remain on the differential diagnosis in patients presenting with persistent or unexplained abdominal pain following hepatic biopsy, even in the absence of portal hypertension or its associated complications. This case highlights the atypical presentation of a centrally located, type 2 APF without the expected manifestations of portal hypertension, demonstrating that the absence of classic clinical findings does not exclude a potentially significant hepatic vascular complication. This case underscores the importance of maintaining a high index of suspicion for APF after hepatic instrumentation and highlights the role of early recognition and multidisciplinary management in achieving favorable clinical outcomes.
